# Hfq regulates antibacterial antibiotic biosynthesis and extracellular lytic-enzyme production in *Lysobacter enzymogenes* OH11

**DOI:** 10.1111/1751-7915.12246

**Published:** 2015-02-13

**Authors:** Gaoge Xu, Yuxin Zhao, Liangcheng Du, Guoliang Qian, Fengquan Liu

**Affiliations:** 1 College of Plant Protection, Nanjing Agricultural University, China/Key Laboratory of Integrated Management of Crop Diseases and Pests (Nanjing Agricultural University), Ministry of EducationNanjing, 210095, China; 2 Department of Chemistry, University of Nebraska-LincolnLincoln, NE, 68588, USA; 3 Institute of Plant Protection, Jiangsu Academy of Agricultural ScienceNanjing, 210014, China

## Abstract

*Lysobacter enzymogenes* is an important biocontrol agent with the ability to produce a variety of lytic enzymes and novel antibiotics. Little is known about their regulatory mechanisms. Understanding these will be helpful for improving biocontrol of crop diseases and potential medical application. In the present study, we generated an *hfq* (encoding a putative ribonucleic acid chaperone) deletion mutant, and then utilized a new genomic marker-free method to construct an *hfq*-complemented strain. We showed for the first time that Hfq played a pleiotropic role in regulating the antibacterial antibiotic biosynthesis and extracellular lytic enzyme activity in *L. enzymogenes*. Mutation of *hfq* significantly increased the yield of WAP-8294A2 (an antibacterial antibiotic) as well as the transcription of its key biosynthetic gene, *waps1*. However, inactivation of *hfq* almost abolished the extracellular chitinase activity and remarkably decreased the activity of both extracellular protease and cellulase in *L. enzymogenes*. We further showed that the regulation of *hfq* in extracellular chitinase production was in part through the impairment of the secretion of chitinase A. Collectively, our results reveal the regulatory roles of *hfq* in antibiotic metabolite and extracellular lytic enzymes in the underexplored genus of *Lysobacter*.

## Introduction

*Lysobacter* is a genus in the family of Xanthomonadaceae and is one of the most ubiquitous environmental microorganisms (Christensen and Cook, 1978). *Lysobacter enzymogenes* of the genus is the best characterized species. This species is known for its ability to produce a variety of extracellular lytic enzymes, including chitinase, cellulase and protease (Kobayashi *et al*., 2005). These enzymes are able to destroy the cell wall of pathogenic fungi and oomycetes and are highly linked to biocontrol activity of *L. enzymogenes* against crop pathogens (Zhang and Yuen, 2000; Palumbo *et al*., 2003). Previously, efforts had been devoted to cloning the genes encoding the lytic enzymes (Epstein and Wensink, 1988; Zhang and Yuen, 2000; Palumbo *et al*., 2003), but little is known about the regulation of the lytic-enzyme production or secretion in *L. enzymogenes*, except the global regulator Clp (Kobayashi *et al*., 2005; Wang *et al*., 2014).

In addition to producing abundant lytic enzymes, *L. enzymogenes* produces diverse bioactive natural products and is recently emerging as a new source of antibiotics, such as the antibacterial WAP-8294A2 (Kato *et al*., 1998; Zhang *et al*., 2011; Xie *et al*., 2012). WAP-8294A2 is a cyclic lipodepsipeptide with a strong activity against Gram-positive bacteria, including methicillin-resistant *Staphylococcus aureus* (Zhang *et al*., 2011). However, *L. enzymogenes* produces a low yield of this antibiotic under the common growth condition (Zhang *et al*., 2011). Meanwhile, the complex chemical structure of WAP-8294A2 makes it extremely challenging for chemical synthesis. We have recently identified the gene cluster responsible for the biosynthesis of WAP-8294A2 in *L. enzymogenes* (Zhang *et al*., 2011). Among the gene, *waps1*, encoding a typical non-ribosomal peptide synthetase, is a key gene for WAP-8294A2 biosynthesis (Zhang *et al*., 2011). The molecular biosynthetic mechanism for WAP-8294A2 production has been proposed (Zhang *et al*., 2011). Little is known, however, about the molecular mechanisms that regulate the biosynthesis of this antibiotic. Understanding the regulatory mechanism for the production of the antibiotic WAP-8294A2 and the lytic enzymes (e.g. chitinase) is important because it could lead to new genetic approaches to improvement of biocontrol of crop disease and potential medical application.

Hfq is a protein serving as a conserved ribonucleic acid (RNA) chaperon and was first characterized as a host factor for phage Qβ RNA replication (Franze de Fernandez *et al*., 1968). It is widely distributed in bacterial annotated genomic databases (Caswell *et al*., 2012; Wang *et al*., 2012). Hfq can bind AU-rich sequence of target messenger RNA (mRNA) and facilitate the pairing interaction between mRNA and small RNA (Wang *et al*., 2012), which suggests that this protein is a global post-transcriptional regulator in most cases. However, recent studies also showed that Hfq was able to directly bind deoxyribonucleic acid (DNA) (Updegrove *et al*., 2010; Geinguenaud *et al*., 2011), even tRNA (Lee and Feig, 2008) and proteins (Butland *et al*., 2005) to modulate the transcriptional expression of target genes, which indicates Hfq also can function as a transcriptional regulator in some bacterial species. Hfq was shown to play critical roles in diverse animal bacterial pathogens and plant-associated bacteria, such as *Escherichia coli*, *Salmonella*, *Sinorhizobium meliloti*, *Staphylococcus* and *Pseudomonas*; the *hfq* mutant exhibited pleiotropic phenotypes in these bacterial species, including decreased growth rate (Fantappiè *et al*., 2009; Chambers and Bender, 2011), increased sensitivity to various environmental stressors (Kadzhaev *et al*., 2009; Schiano *et al*., 2010) and attenuated virulence (Geng *et al*., 2009; Ramos *et al*., 2011). The role of *hfq* in a number of bacterial biological control agents had been investigated. For example, Hfq was found to regulate antibiotic production in *Pseudomonas aeruginosa* M18, a rhizobacterium bacterium that can efficiently inhibit soil-borne phytopathogenic fungi (Wang *et al*., 2012). In *Pseudomonas fluorescens* 2P24, *hfq* is involved in the colonization, biofilm formation, antibiotic synthesis and quorum sensing signal production (Wu *et al*., 2010). However, essentially nothing is known about the role of *hfq* in any of *Lysobacter* species.

In the present study, we identified an *hfq* homologue from the genome of strain OH11, a Chinese isolate of *L. enzymogenes*. The results show that this *hfq* plays a pleiotropic role in regulating the antibacterial antibiotic biosynthesis and extracellular lytic enzyme activity in *L. enzymogenes*. To our knowledge, this study represents the first attempt to address the regulatory function of *hfq* in the genus of *Lysobacter*. Our findings also add an understanding of the conserved Hfq protein in different bacterial species.

## Results and discussion

### Sequence analysis of hfq in L. enzymogenes

**Fig 1:**
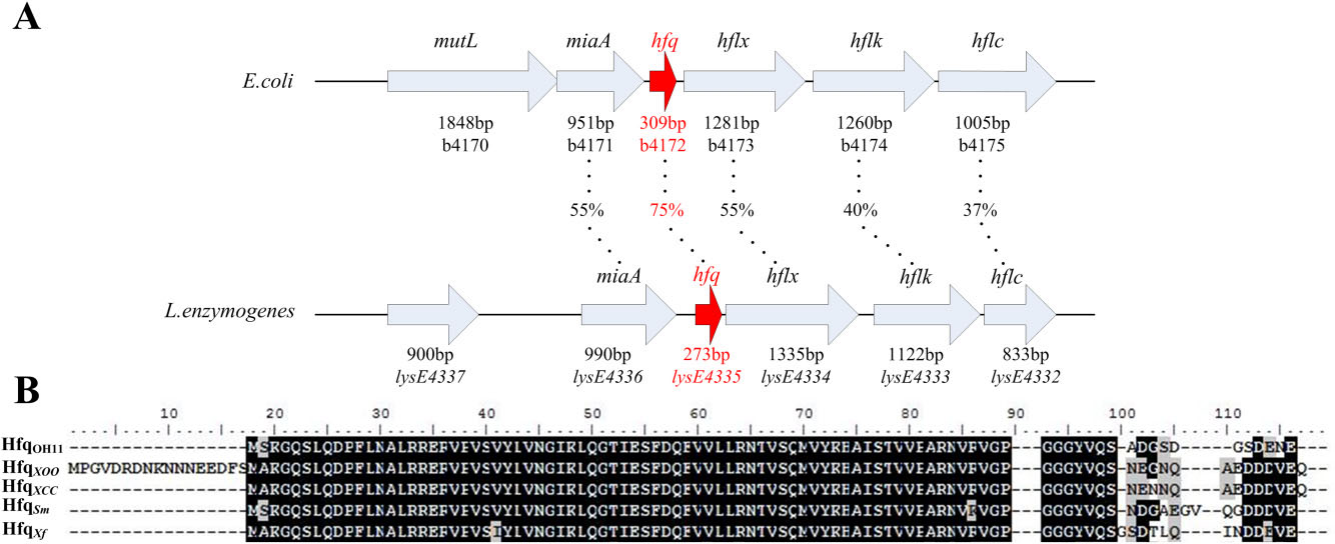
Identification of Hfq (LysE4335) in *Lysobacter enzymogenes*.A. Comparison of the *hfq* locus between *L*. *enzymogenes* strain OH11 and the well-studied *Escherichia coli*. The percentage numbers (expressed by %) represent the identity/similarity between the gene/protein homologue in *E. coli* and *L*. *enzymogenes* at amino-acid level. The size of each gene was presented below each arrow.B. Sequence alignment of Hfq_OH11_ of *L*. *enzymogenes* with other Hfq proteins from taxonomically related bacterial species, all belonging to the Xanthomonadaceae family.

Hfq protein was first discovered from *E. coli*, and this Hfq was considered as the model in bacteria (Franze de Fernandez *et al*., 1968; Kajitani and Ishihama, 1991). To examine whether *L. enzymogenes* possesses an Hfq homologue, we used the *E. coli* Hfq protein (AAC43397.1) as the query sequence to perform a local BlastP search in the draft genome of strain OH11 (Lou *et al*., 2011). This led to the identification of an Hfq homologue (KM186922) in *L. enzymogenes*. As shown in Fig. 1, Hfq protein of *L. enzymogenes* shares 75% similarity to that of *E. coli* at the amino acid level. Moreover, the locus of *miaA-hfq-hflX-hflK-hflC* of *E. coli* was also conserved in *L. enzymogenes*. Next, we selected the reported Hfq proteins from four *Lysobacter*-related bacterial species, all belonging to the Xanthomonadaceae family, to do a sequence alignment. The result showed that Hfq of *L. enzymogenes* shared a high similarity (85–96%) to that of these taxonomically related species, including Hfq*_xoo_* of *Xanthomonas oryzae* pv*. oryzae* (WP_014503655.1), Hfq*_xcc_* of *X. campestris* pv*. campestris* (WP_011036893.1), Hfq*_sm_* of *Stenotrophomonas maltophilia* (WP_019183319.1) and Hfq*_xf_* of *Xylella fastidiosa* (WP_004085558.1). The results suggest that *L. enzymogenes* possesses a putative conserved Hfq protein.

### Generation of a set of marker free of hfq-derived strains of L. enzymogenes

**Fig 2:**
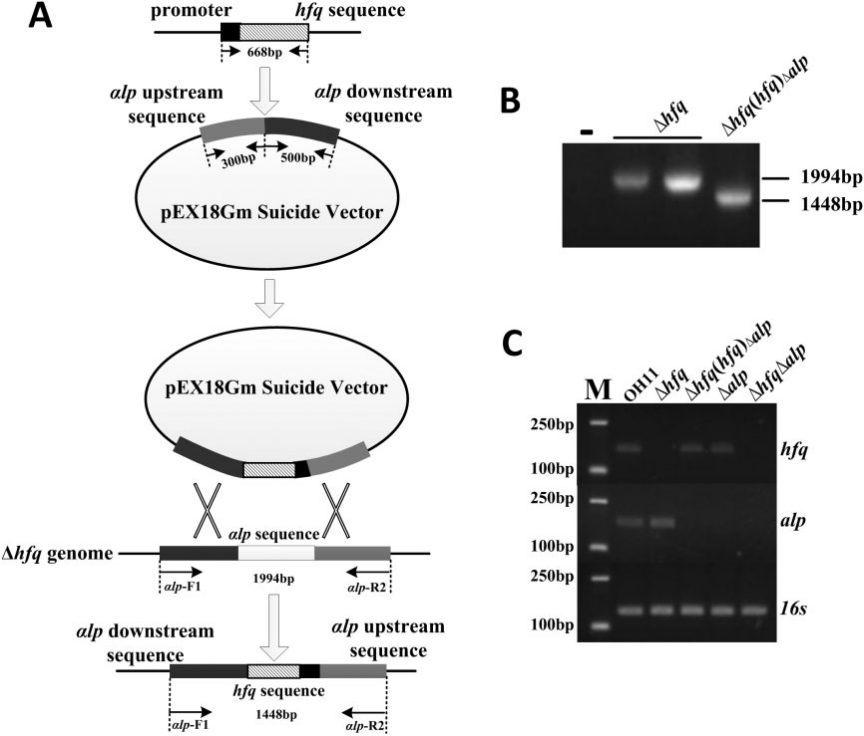
The marker-free integration of *hfq* to generate a complementation strain of *Lysobacter enzymogenes*.A. Physical map of the marker-free complemented strain of the *hfq* mutant.B. PCR confirmation of the replacement of the *αlp* gene by *hfq*.C. RT-PCR to conform the expression of the target gene (*hfq* or *αlp*) in the *hfq* mutant and its derivative strains. OH11, the wild-type strain of *L*. *enzymogenes*; Δ*hfq*, the *hfq* deletion mutant; Δ*hfq*(*hfq*)_Δ_*_αlp_*, the genomic marker-free complemented strain of the *hfq* mutant (the *αlp* gene was replaced by the *hfq* in the background of the *hfq* mutation); Δ*αlp*, the *αlp* deletion mutant; Δ*hfq*Δ*αlp*, the double mutant of *hfq* and *αlp*. *αlp*, an α-lytic-protease encoding gene of *L*. *enzymogenes*. ‘-’ in B represents the blank control.

In order to explore the function of *hfq* in *L. enzymogenes*, a 155-bp internal fragment of *hfq* was in-frame deleted, which led to the generation of an *hfq* mutant, named as Δ*hfq* (Fig. S1). Subsequently, a genomic marker-free strategy was utilized to construct the *hfq* complemented strain (Fig. 2A). In this *hfq* complemented strain, no exogenous antibiotic selection markers were introduced into the *hfq* mutant, which can eliminate their potential effect on the tested phenotypes of the present study in *L. enzymogenes*. For this purpose, we selected *αlp* (Wang and Qian, 2012), an α-lytic-protease encoding gene as a target for *hfq* integration. To our knowledge, this gene is not associated with the tested phenotypes of the present study, including the antibiotic WAP-8294A2 production and the activity of extracellular chitinase, protease or cellulase. By using homologous recombination method as shown in Fig. 2A, we introduced the intact *hfq* gene together with its own promoter into the *αlp* genomic locus in the background of the *hfq* mutant, and generated a chromosomal marker-free *hfq*-complemented strain Δ*hfq*(*hfq*)_Δ_*_αlp_* (Fig. 2B). Meanwhile, we also created single mutation of *αlp* (Δ*αlp*) and double mutations of *hfq* and *αlp* (Δ*hfq*Δ*αlp*) as controls (Table S1). To verify the mutants, we utilized an reverse transcription polymerase chain reaction (RT-PCR) assay to examine whether the target gene (*hfq* or *αlp*) was transcribed or not in these *hfq*-derived strains. As shown in Fig. 2C, we detected the transcription of *hfq* in wild-type OH11, the *hfq*-complemented strain (Δ*hfq*(*hfq*)_Δ_*_αlp_*) and the *αlp* mutant (Δ*αlp*) but not in the *hfq* mutant (Δ*hfq*) and the double mutant (Δ*hfq*Δ*αlp*). Similarly, it was also found that *αlp* was transcribed in wild-type OH11 and the *hfq* mutant (Δ*hfq*) but not in the *hfq* complemented strain, the *αlp* deletion mutant and the double mutant. These results verified the *hfq*, *αlp*, double mutations and the marker-free *hfq* complemented strains.

### Mutation of hfq caused a significant increase of the yield of WAP-8294A2 in L. enzymogenes

**Fig 3:**
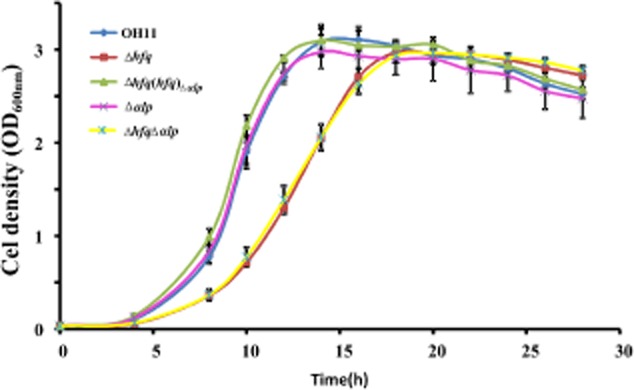
The growth curves of various *Lysobacter* strains in 20% TSB medium. The growth level of each strain was measured by OD_600 nm_ at regular intervals (2 h or 4 h). Three replicates for each treatment/strain were used, and the experiment was performed three times. Vertical bars represent standard errors. The strain information in Fig. 3 is shown in the legend of Fig. 2.

To test the role of *hfq* in the regulation of the antibiotic WAP-8294A2 biosynthesis, we examined WAP-8294A2 production in the *hfq* mutant in *L. enzymogenes*. To eliminate the potential influence of growth alteration on WAP-8294A2 production between the wild-type strain and the *hfq* mutant, we subsequently determined the growth ability (expressed by OD_600 nm_, the optical density at 600 nm) of wild-type OH11, the *hfq* mutant and its derivative strains in 20% TSB broth that was used to cultivate these *Lysobacter* strains for WAP-8294A2 extraction. Meanwhile, considering that inactivation of *hfq* in several bacteria leads to an increased cell size (Tsui *et al*., 1994; Boudry *et al*., 2014), we then tested the role of *hfq* in this phenotype (cell size) in *L. enzymogenes*, because if disruption of *hfq* causes bigger cells, it will affect the cell density that is expressed by OD_600 nm_ in the present study. As shown in Fig. S2, we found that mutation of *hfq* almost did not alter cell size compared with the wild-type strain at the two selected time points (after growth of 24 h and 48 h). These results suggest that application of OD_600 nm_ as an indicator to reflect the cell growth status between the wild-type strain and the *hfq* mutant should be reasonable in the present study. Next, the growth rate of the wild-type strain and the *hfq* mutant in 20% Tryptic Soy Broth (TSB) broth was determined and compared. As shown in Fig. 3, we observed that mutation of *hfq* resulted in a changed growth pattern that was taken place in the logarithmic phase and originated the delay in reaching the stationary phase compared with that of the wild-type strain. Under the same condition, the complemented strain exhibited the wild-type growth rate. As expected, mutation of *αlp* did not alter the wild-type growth rate, indicating that *αlp* was not involved in the growth capacity in the tested medium. This finding was further verified by the growth rate of the double mutant (Δ*hfq*Δ*αlp*), as this double mutant displayed a closely similar growth rate to that of the *hfq* mutant. The results suggested that mutation of *hfq* had a slight effect on the growth of the wild-type strain in the tested medium (20% TSB).

**Fig 4:**
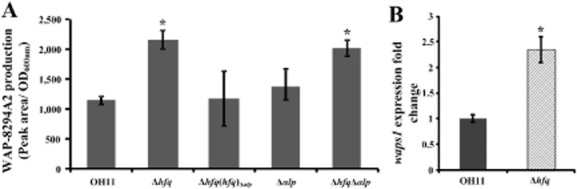
Mutation of *hfq* significantly increased the production of WAP-8294A2 in *Lysobacter enzymogenes*.A. Quantitative measurement of the yield of the antibacterial antibiotic WAP-8294A2 in the *hfq* mutant and its derivative strains.B. Quantitative determination of the transcription of the critical biosynthetic gene (*waps1*) for WAP-8294A2. The strain information in Fig. 4 is shown in the legend of Fig. 2. Each column indicates the mean of three biologically independent experiments. Vertical bars represent standard errors. ‘*’ (*P* < 0.05; *t*-test) above the bars indicate a significant difference between the wild-type strain OH11 and the *hfq* mutant.

Next, we cultivated the tested *Lysobacter* strains in 20% TSB broth to extract WAP-8294A2, and determined its yield in each strain by high-performance liquid chromatography (HPLC). To completely eliminate the influence of growth alteration on WAP-8294A2 production, we used the indicator of ‘Antibiotic production (peak area/OD_600 nm_)’ to quantitatively evaluate the ability of WAP-8294A2 production in the wild-type strain and its derivatives. Here, peak area indicates the area of WAP-8294A2 determined by HPLC, whereas OD_600 nm_ represents the cell density of the tested strains at the time point used for the extraction of WAP-8294A2 in *L. enzymogenes*. In this way, we found that mutation of *hfq* significantly enhanced WAP-8294A2 production (∼ 2.2 fold) compared with the wild-type strain, whereas the marker-free complemented strain of the *hfq* mutant displayed the wild-type level in this ability (Fig. 4A). Given that the marker-free *hfq* complemented strain was constructed by integration of *hfq* into the *αlp* gene in the background of *hfq* mutation, the parent *hfq* and *αlp* genes were both missing in this complemented strain. Therefore, it is also possible that the restoration of WAP-8294A2 production in the marker-free *hfq* complemented strain could be due to the effect of both missing *hfq* and *αlp*. To test this possibility, we determined the WAP-8294A2 production in the double mutant (Δ*hfq*Δ*αlp*). We showed that mutation of *αlp* in the background of the *hfq* mutant did not affect the yield of WAP-8294A2 in this mutant (the *hfq* mutant) (Fig. 4A). This result provided supportive evidence to verify that restoration of WAP-8294A2 production in the marker-free *hfq* complemented strain was due to the replacement of *αlp* by *hfq* in the background of the *hfq* mutation but not associated with the effect of the simultaneous deletion of both *hfq* and *αlp*. Furthermore, mutation of *αlp* in the background of wild-type strain did not alter WAP-8294A2 production, supporting that *αlp* was not associated with WAP-8294A2 biosynthesis. Collectively, these results indicated that *hfq* played an important role in the negative regulation of WAP-8294A2 biosynthesis in *L. enzymogenes*.

To further verify the important role of *hfq* in WAP-8294A2 biosynthesis, the transcriptional level of *waps1* (Zhang *et al*., 2011), the key gene responsible for WAP-8294A2 biosynthesis, was tested using quantitative (q)RT-PCR between the wild-type strain and the *hfq* mutant. Based on the result of Fig. 3, we finally collected the cells at the logarithmic phase from the wild-type strain and the *hfq* mutant at different time point corresponds to the same cell density (OD_600 nm_ = 1.0), respectively, because at this cell density, the gene *waps1* was previously shown to be expressed at transcriptional level in the wild-type OH11 of *L. enzymogenes* (Wang *et al*., 2014). The results of the qRT-PCR assay showed that mutation of *hfq* caused a significant increase (∼ 2.3 fold) of the transcription of *waps1* compared with the wild-type strain (Fig. 4B). This finding was consistent with the HPLC result for the WAP-8294A2 production in the *hfq* mutant (Fig. 4A), and further verified that *hfq* was involved in the negative regulation of WAP-8294A2 biosynthesis in *L. enzymogenes*.

### Mutation of hfq almost abolished extracellular chitinase activity and significantly reduced the activity of both extracellular protease and cellulase in L. enzymogenes

**Fig 5:**
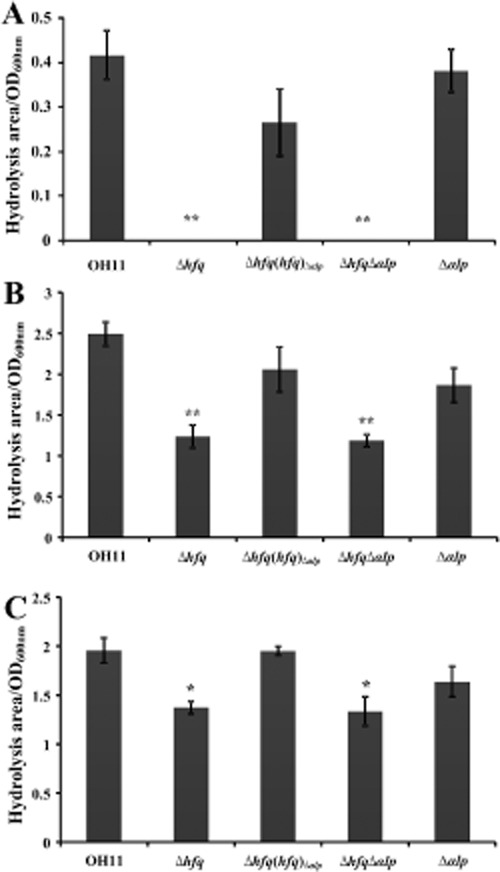
Quantitative determination of the activity of three extracellular lytic enzymes, chitinase (A), protease (B) and cellulase (C) from various *Lysobacter enzymogenes* strains. Each column indicates the mean of three biologically independent experiments. Vertical bars represent standard errors. ‘*’ (*P* < 0.05; *t*-test) or ‘**’ (*P* < 0.01; *t*-test) above the bars indicate a significant difference between the wild-type strain and its derivatives. The strain information in Fig. 5 is shown in the legend of Fig. 2.

In addition to WAP-8294A2 biosynthesis, we are also interested in addressing the role of *hfq* in lytic-enzyme production in *L. enzymogenes*. We therefore examined the activity of three known extracellular lytic enzymes, including chitinase, protease and cellulase on the corresponding detecting media. In the present study, the ratio of the area of hydrolytic zones divided by cell density (OD_600 nm_) was used as a quantitative indicator for each enzyme activity. By this way, we found that mutation of *hfq* almost abolished extracellular chitinase activity, and significantly reduced the activity of both extracellular protease (*P* < 0.01; *t*-test) or cellulase (*P* < 0.05; *t*-test) in *L. enzymogenes* (Fig. 5). Under the same conditions, the marker-free *hfq* complemented strain restored the wild-type level in each tested enzyme activity (Fig. 5). Furthermore, mutation of *αlp* in the background of the *hfq* mutant did not influence the activity of each tested enzymes (Fig. 5). This result suggested that the activity restoration of each tested enzymes in the marker-free *hfq* complemented strain was due to the replacement of *αlp* by *hfq* in the background of the *hfq* mutation but not associated with the effect of the deletion of both *hfq* and *αlp*. Moreover, mutation of *αlp* in the background of the wild-type strain did not alter the activity of each tested enzyme (Fig. 5), supporting that *αlp* was not involved in the regulation of the activity of these three tested enzymes in *L. enzymogenes*. Collectively, our results indicated that *hfq* played a key role in the regulation of the activity of extracellular chitinase, protease and cellulase in *L*. *enzymogenes*.

### Mutation of hfq inhibited the secretion of chitinase A

The deficiency of the *hfq* mutant in extracellular chitinase production promotes us to focus the mechanism(s) by which *hfq* modulates this activity in *L. enzymogenes*. For this purpose, we first investigated whether the deficiency of the *hfq* mutant in extracellular chitinase activity might be associated with its growth reduction, as we observed that the final cell density of the *hfq* mutant was significantly reduced (∼ 2.3 fold) compared with that of the wild-type strain when the initial inoculated cell concentration was the same (Fig. S3). Subsequently, the experimental evidence presented here eliminated this possibility, because we clearly found that even though the *hfq* mutant possessed a significantly increased cell density (*P* < 0.05; *t*-test) compared with the wild-type strain (when the initial inoculated cell concentration of the *hfq* mutant was 10-fold higher than that of the wild-type strain) (Fig. S3), we still did not find any hydrolytic zone around the colonies of the *hfq* mutant (Fig. S3). These results provided supportive evidence to show that the deficiency of the *hfq* mutant in chitinase activity was not due to its growth reduction, which implies that other mechanism(s) may be utilized by *hfq* to modulate extracellular chitinase production in *L. enzymogenes*.

Next, we attempted to test which type(s) of chitinases was reduced in the *hfq* mutant, resulted in its deficiency in extracellular chitinase activity. For this purpose, we made a survey to detect how many chitinase-encoding genes are present in the genome of *L. enzymogenes*. This led to identification of a total of three chitinase-encoding genes, including *chiA* (Qian *et al*., 2012), *chiB* and *chiC* (Table S3). We subsequently generated the *chiB* and *chiC* deletion mutants (Table S1). The extracellular chitinase activity of these two mutants as well as the *chiA* mutant (Qian *et al*., 2012) was then tested. As shown in Fig. S4, we found that mutation of *chiA* completely abolished the chitinase activity, which was closely consistent with the role of the *hfq* mutant in this phenotype (Fig. 5). However, mutation of *chiB* or *chiC* did not alter the extracellular chitinase activity (Fig. S4). This finding raised the possibility that the regulation of *hfq* on extracellular chitinase activity was probably through the alteration of *chiA* in *L. enzymogenes* under the testing conditions.

**Fig 6:**
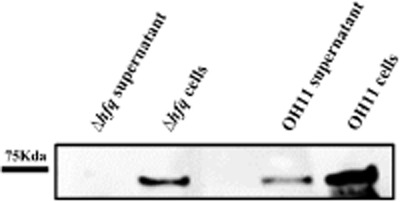
Western blot analysis of the yield of flag-tagged ChiA (Chitinase A) in *Lysobacter enzymogenes*. The yield of flag-tagged ChiA (Chitinase A) both in the supernatant and total cell of the wild-type strain OH11 and the *hfq* mutant (Δ*hfq*) was comparatively analysed by western blot using the anti-flag antibody. The data are the representative results of three independent experiments. The expected size of ChiA protein is 71.6 Kda.

Considering the fact that Hfq usually functions as a post-transcriptional regulator (Lee and Feig, 2008; Sittka *et al*., 2009), we then examined whether *hfq* plays a post-transcriptional regulation on *chiA*. We first constructed a plasmid (pBBR1-MCS5)-based *hfq*-complemented strain by introduction of pBBR1-MCS5 containing the *hfq* gene into the *hfq* mutant. Then, the extracellular chitinase activity was tested in this complemented strain. We showed that the plasmid (pBBR1-MCS5)-based *hfq*-complemented strain partially restored the wild-type level in extracellular chitinase activity, whereas the *hfq* mutant containing the empty vector was deficient in this function (Fig. S5). This result indicates that the plasmid (pBBR1-MCS5) containing *hfq* was functional in restoring the deficiency of the *hfq* mutant in extracellular chitinase activity. Next, a flag-tagged *chiA* sequence was cloned into the broad-host-vector pBBR1-MCS5, and transformed into the *chiA* mutant. The introduction of pBBR1-MCS5 containing the flag-tagged *chiA* restored the chitinase activity of the *chiA* mutant (Fig. S6A), supporting the correction of the construction. We then individually introduced the pBBR1-MCS5 containing the flag-tagged *chiA* into the *hfq* mutant and wild-type OH11, respectively. Next, the anti-flag antibody dependent western blot assay was performed to compare the protein content of the flag-tagged ChiA (Chitinase A) in wild-type OH11 and the *hfq* mutant. As shown in Fig. 6, we observed that the band of flag-tagged ChiA was detected both in the total cells and the supernatant of wild type, suggesting ChiA was synthesized and secreted outside the cells under the testing condition. Meanwhile, we only detected the flag-tagged ChiA band in the total cells but not in the supernatant of the *hfq* mutant. This result indicated the ChiA protein was probably synthesized in the cells of the *hfq* mutant but was unable to be secreted out of the cells or degraded once secreted in the *hfq* mutant. To verify this, we introduced the construct (pBBR1-MCS5 containing the flag-tagged *chiA*) into the *hfq* mutant and tested its chitinase activity. We found that the *hfq* mutant containing this construct did not restore the chitinase activity, as no hydrolytic zones around its colonies was detected on the chitinase selective medium, whereas the *chiA* mutant and wild-type OH11 containing this construct displayed visible chitinase activity (Fig. S6B). This result provided supportive evidence to show that the effect of *hfq* on chitinase activity was probably in part through the impairment of the secretion of ChiA in *L. enzymogenes*. However, we do not know how *hfq* mediates the secretion of ChiA in *L. enzymogenes* at this time.

A recent study shows that in *Flavobacterium johnsoniae* chitinase secretion is dependent on type IX secretion system (T9SS), consisting of products of the key genes of *gldK*, *gldL*, *gldM*, *gldNO*, *sprA*, *sprE* and *sprT* (Kharade and McBride, 2014). However, we did not find the homologue of any of these T9SS-associated key genes/proteins in the genome of *L. enzymogenes* OH11, indicating that the effects of *hfq* on the secretion of Chitinase A in *L. enzymogenes* is probably not associated with T9SS and is therefore different from the finding in *F. johnsoniae*. Similar to our result, the *hfq* mutant of *Listeria monocytogenes* showed a less chitinolytic activity compared with that of wild-type strain. This report pointed out that uncharacterized *hfq*-dependent small RNAs may mediate the stimulating effect of *hfq* on the activity and/or secretion of the chitinase in *L. monocytogenes* (Nielsen *et al*., 2011). This information provided a clue to further explore the regulatory mechanism of *hfq* on the secretion of chitinase A by focusing on the *hfq*-dependent small RNAs in *L. enzymogenes*.

### Conclusion remarks

The production of lytic enzymes and antibiotics is one of the distinctive characteristics of the important but underexplored biological control agent *L. enzymogenes*. The genetic determinants that regulate the biosynthesis of these factors are largely unidentified. The present study reported Hfq, a putative RNA chaperone played a pleiotropic role in the modulation of the antibacterial antibiotic WAP-8294A2, and the activity of extracellular chitinase, protease and cellulase in *L. enzymogenes* OH11. The regulation of *hfq* on extracellular chitinase production was further shown to be in part through the impairment of the secretion of chitinase A. These findings provide new insights into the role of *hfq* in bacteria. In future works, we aim to further address the molecular mechanisms of *hfq* on the regulation of WAP-8294A2 as well as the secretion of chitinase A in *L. enzymogenes*. These future works will help us further understand the signalling pathway of *hfq* in the regulation of antibiotics and lytic enzymes in *L. enzymogenes*.

## Experimental procedures

### Bacterial strains and growth conditions

Strains and plasmids used in this study are shown in Table 1. *Escherichia coli*, strain Top 10 was grown in LB medium at 37°C. Unless otherwise stated, *Lysobacter enzymogenes* strain OH11 (CGMCC No. 1978), and its derivative strains were grown in LB medium at 28°C. When required, appropriate antibiotics were added into the medium to a final concentration of kanamycin (Km) 50 μg ml^−1^ and gentamicin (Gm) 150 μg ml^−1^.

**Table 1:** Strains and plasmids used in this study

Strains and plasmids	Characteristics^a^	Source
*Lysobacter enzymogenes*		
OH11	Wild-type, Km^R^	Qian *et al*., 2009
Δ*hfq*	*hfq* in-frame deletion mutant, Km^R^	This study
Δ*hfq*(*hfq*)	Δ*hfq* harbouring plasmid *hfq*-pBBR, Gm^R^, Km^R^	This study
Δ*hfq*(pBBR)	Δ*hfq* harbouring plasmid pBBR1-MCS5, Gm^R^, Km^R^	This study
Δ*hfq*Δ*αlp*	*hfq* and αlp in-frame deletion mutant, Km^R^	This study
Δ*hfq*(*hfq*)_Δ_*_αlp_*	*hfq* in-frame deletion mutant and the *αlp* gene was replaced by *hfq*, Km^R^	This study
Δ*αlp*	*αlp* in-frame deletion mutant, Km^R^	Wang and Qian, 2012
Δ*chiB*	*chiB* in-frame deletion mutant, Km^R^	This study
Δ*chi*C	*chiC* in-frame deletion mutant, Km^R^	This study
Δ*chiA*	*chiA* in-frame deletion mutant, Km^R^	Qian *et al*., 2012
Δ*chiA*(*chiA*-flag)	Δ*chiA* harbouring flag-tagged *chiA*-pBBR	This study
OH11(*chiA*-flag)	OH11 harbouring flag-tagged *chiA*-pBBR	This study
Δ*hfq*(*chiA*-flag)	Δ*hfq* harbouring flag-tagged *chiA*-pBBR	This study
*Escherichia coli*		
TOP10	*supE44lacU169*(Δ*lacZ*ΔM15) *hsdR17 recA lendA1gyrA96 thi-1 relA11*	Lab collection
Plasmids		
pEX18GM	Suicide vector with a *sacB* gene, Gm^R^	Hoang *et al*., 1998
pBBR1-MCS5	Broad-host- vector with a P*_lac_* promoter, Gm^R^	Kovach *et al*., 1995
*hfq*-pEX18	pEX18GM with two flanking fragments of *hfq*, Gm^R^,	This study
*αlp*-pEX18	pEX18GM with two flanking fragments of *αlp*, Gm^R^,	Wang and Qian, 2012
*hfq*-pBBR	pBBR1-MCS5 cloned with a 668-bp fragment containing intact *hfq* and its predicted promoter	This study
*chiB*-pEX18	pEX18GM with two flanking fragments of *chiB*, Gm^R^,	This study
*chiC*-pEX18	pEX18GM with two flanking fragments of *chiC*, Gm^R^,	This study
*chiA*-flag-pBBR	pBBR1-MCS5 cloned with a 2403 bp fragment containing flag-tagged *chiA*	This study

a Km^R^, Gm^R^ = Kanamycin, Gentamicin-resistance respectively.

### Generation of deletion mutants of target genes in L. enzymogenes

The wild-type strain OH11 (CGMCC No. 1978) of *L. enzymogenes* was used as an original strain to generate the in-frame deletion mutants. Construction of gene-deletion mutants in *L. enzymogenes* was performed as described previously (Qian *et al*., 2013). As a representative example, the scheme of the *hfq* mutant construction and molecular confirmation in *L. enzymogenes* was provided in Fig. S1 in the supplementary file. All the primers and plasmids used in the present study were provided in Table S2 and Table 1 respectively.

### Construction and confirmation of a marker-free hfq complemented strain

The *hfq* fragment with its predicted promoter (668-bp) was inserted into a pEX18Gm-*αlp* (Wang and Qian, 2012) recombinant suicide vector, which has an upstream fragment (300-bp) and a downstream fragment (500-bp) of *αlp* gene of the wild-type OH11. The final construct was transformed into the *hfq* mutant. After twice homologous recombination, the *αlp* gene was replaced by *hfq* in the genome of the wild-type OH11, which is defined as the marker-free-complemented strain of the *hfq* mutant. This marker-free *hfq*-complemented strain was verified both by PCR and RT-PCR assays. For PCR assay, the expected size of the DNA fragment from the *hfq* mutant was 1994 bp by using the primers of *αlp*-F1/R2 (Table S2). When the *αlp* gene was replaced by the *hfq* gene in the background the *hfq* mutant, the expected size of DNA fragment was 1448 bp by using the same primer pairs (*αlp*-F1/R2). For RT-PCR assay, the expression of the target gene (*hfq* or *αlp*) in the *hfq* mutant and its derivative strains was tested. The expected size of the DNA fragment amplified from the complementary DNA (cDNA) of the wild-type strain corresponds to the *hfq*, *αlp* and 16s rRNA was 158 bp, 174 bp and 147 bp respectively. The corresponding primers were shown in Table S4. In the present study, 16s rRNA was used as an internal control as described previously (Qian *et al*., 2013; 2014).

### Observation of the cell size of L. enzymogenes by electronic microscope

The wild-type OH11 and the *hfq* deletion mutant were grown on 20% TSB with agar (TSA) plates, and their cells were collected after growth of 24 h and 48 h, respectively. These cells were further used for the analysis of electronic microscope as described previously in our lab (Chen *et al*., 2009)

### Detection of growth curve

Various *Lysobacter* strains were cultured in LB medium at 28°C overnight. Then, 500 μl of the overnight culture for each strain was transferred into the fresh 20% TSB broth (50 ml) to grow until the cell density expressed by OD_600 nm_ reached to 1.0. Next, 1 ml of each culture was transferred again into the fresh 20% TSB broth (50 ml) to start the detection of growth curve. All inoculation broths were grown at 28°C with shaking at 200 r.p.m., and the OD_600 nm_ value was determined every 2 h or 4 h until bacterial growth reached the stationary stage. Each sample involves three technical replicates and the experiment was performed three times.

### RNA extraction, qRT-PCR and RT-PCR

The wild-type OH11 and the *hfq* deletion mutant were grown on 20% TSB. The cells of the wild-type strain and the *hfq* mutant were collected at the time point, 8 h and 11 h, corresponds to the same cell density (OD_600 nm_ = 1.0). Then, the total RNA was extracted from the cells of each strain using a kit with a code of R6950-01 from OMIGA Company (China). Next, the qRT-PCR and RT-PCR assays, including cDNA synthesis and PCR amplification were performed as described previously (Qian *et al*., 2013; 2014). Primer sequences used in this assay are listed in Table S4.

### Extraction and detection of WAP-8294A2

Various *Lysobacter* strains were cultured in 20% TSB broth at 28°C until the cell density expressed by OD_600 nm_ reached to 1.0. Next, 1 ml of each culture was transferred into the fresh 20% TSB broth (50 ml) for 3 days shaking culturing. Then, the extraction and HPLC analysis of WAP-8294A2 from *L. enzymogenes* were performed as described previously (Zhang *et al*., 2011; 2014). Three replicates were used for each strain, and the experiment was performed three times.

### Quantitative determination of the activity of three extracellular lytic enzymes

A sterile filter membrane (10 mm diameter) was put on the surface of the selective plates for chitinase, protease and cellulase. The composition of each selective plates was described in previous studies (Kobayashi *et al*., 2005; Qian *et al*., 2013). In brief, 3 μl of bacterial culture of various *Lysobacter* strains with the same cell density (OD_600 nm_ = 2.0) was spotted on the filter membrane. After 3 days of growth, the filter member was taken off from the plates. The diameters of the hydrolytic zones in the plates were measured and the corresponding hydrolytic areas were calculated. Meanwhile, the cells of each strain on the filter member were washed by 800-μL sterilized ddH_2_O, and the cell density of each strain was measured and expressed by OD_600 nm_. Finally, the ratio of the area of hydrolytic zones divided by cell density (OD_600 nm_) was used as a quantitative indicator for each enzyme activity of the tested *Lysobacter* strains.

### Western blot

The flag-tagged *chiA* was amplified by PCR with the primers of *chiA*-F and *chiA*-R (flag) (Table S2), and cloned into the broad-host-vector pBBR1-MSC5 (Table 1). Then, the pBBR1-MCS5 containing the flag-tagged *chiA* was transferred into wild-type OH11 and the *hfq* deletion mutant to generate two strains, OH11 (*chiA*-flag) and Δ*hfq* (*chiA*-flag). These strains were cultured in 50 ml of LB broth to grow until the value of OD_600 nm_ reached to 2.0. Then, the total cells and the culture supernatants of tested strains were collected respectively. Next, for cells, 1-ml RIPA buffer (CWBIO Company, China) with a code of 1713L was used to lyse cells, followed by a centrifugation (10 000 × *g* at 4°C for 10 min). These cell supernatants were collected and used for further study. For culture supernatants, they were concentrated to 1 ml using a vacuum freeze drier, and used for further studies. The following western blot assay was performed as described previously (Ansong *et al*., 2009). The experiments were performed three times, and each involves three replicates for each treatment.

### Data submission

The sequence data of the present study have been submitted to the National Center for Biotechnology Information Genbank under accession number KM186921(*miaA*), KM186922 (*hfq*), KM186923 (*hflx*), KM186924 (*hflk*) KM186925 (*hflc*) and KM186926 (*αlp*) respectively.

### Data analysis

All analyses were conducted using spss 14.0 (spss Inc, Chicago, IL, USA). The hypothesis test of percentages (*t*-test, *P* < 0.05 or 0.01) was used to determine significant differences in the production of antibiotic metabolites, lytic-enzyme activity and gene expressions between the wild-type OH11 and its derivatives.

## Supporting Information

**Fig. S1.** The scheme of the *hfq* mutant construction and molecular confirmation in *Lysobacter enzymogenes*. A. Physical map of deletion mutant construction used in this study. The 364-bp (amplified by *hfq*-F1/R1) (Table S2) and 368-bp (amplified by *hfq*-F2*/*R2) (Table S2) DNA fragment of *hfq* was used as 5′ and 3′ fragment for homologue recombination respectively. The internal 155-bp DNA fragment would be deleted in the *hfq* mutant. The primers *hfq*-F1/R2 (Table S2) was used for molecular confirmation of *hfq* mutant. B. PCR verification of the *hfq* mutant. An 887-bp DNA fragment was amplified from the wild-type OH11 with the primers *hfq*-F1/R2, while only a 732-bp fragment was obtained from the *hfq* mutant with the same primers due to the deletion of internal 155-bp DNA fragment. ‘-’ in B represents the blank control. This strategy was applied into the construction of other mutants of target genes in the present study (Table S1 and Table S2). **Fig. S2.** Comparison of the cell size between the wild-type strain and the *hfq* mutant of *Lysobacter enzymogenes*. A. The representative result of cell size from three independent experiments between the wild-type strain (OH11) and the *hfq* mutant (Δ*hfq*) under electronic microscope at two selected time points (24 h and 48 h after growth on solid 20% TSA medium). The scale bars represent 1 μm. B. Statistic analysis of the cell size of the wild-type strain and the *hfq* mutant. In each technological repeat, at least 15 cells of each strain were selected for analysis. The experiment was performed three times. Each column indicates the mean of three biologically independent experiments. Vertical bars represent standard errors. No significant difference (*P* < 0.05; *t*-test) in cell size between the wild-type strain and the *hfq* mutant was found. **Fig. S3.** Further determination of extracellular chitinase activity between the wild-type strain and the *hfq* mutant of *Lysobacter enzymogenes* on solid medium. A. The representative phenotype of extracellular chitinase activity between the wild-type OH11 and the *hfq* mutant from three independent experiments. B. Quantitative analysis of cell density (OD_600 nm_) of each strain on the surface of the filter membrane. Each column indicates the mean of three biologically independent experiments. Vertical bars represent standard errors. ‘**’ (*P* < 0.01; *t*-test) above the bars indicate a significant difference between the wild-type strain and the *hfq* mutant. The initial inoculated cell concentration expressed by OD_600 nm_ for the wild-type OH11, Δ*hfq*-a, Δ*hfq*-b was 2, 2 and 20 respectively. **Fig. S4.** Detection of the chitinase activity of three mutants of predicted chitinase synthesis genes (Δ*chiA*, Δ*chiB*, Δ*chiC*) on chitin plate. Only the *chiA* mutant cannot hydrolyse chitin under the tested conditions. The gene information of *chiB* and *chiC* was provided in Table S3. The mutant construction and confirmation was provided in Table S1. **Fig. S5.** Introduction of the broad-host-vector pBBR1-MCS5 containing *hfq* partially restored extracellular chitinase production of the *hfq* mutant in *Lysobacter enzymogenes*. OH11, the wild-type strain of *L. enzymogenes*; Δ*hfq*, the *hfq* deletion mutant; Δ*hfq*(pBBR), the *hfq* mutant containing the empty vector (pBBR1-MCS5); Δ*hfq*(*hfq-*pBBR), the pBBR1-MCS5-based *hfq* complemented strain. Figure S5 is the representative result of three independent experiments. **Fig. S6.** Determination of extracellular chitinase activities of *Lysobacter* strains. A. The construct of flag-tagged *chiA* restored the chitinase activity of the *chiA* mutant. B. The *hfq* mutant containing the construct of flag-tagged *chiA* did not restore the chitinase activity. OH11, the wild-type strain of *L. enzymogenes*; Δ*chiA*, the *chiA* deletion mutant (Qian *et al*., 2012); Δ*chiB*, the *chiB* deletion mutant; Δ*chiC*, the *chiC* deletion mutant; Δ*chiA*(*chiA*-flag), the *chiA* mutant containing flag-tagged *chiA*; Δ*hfq*(pBBR), the *hfq* mutant containing an original pBBR1-MSC5 vector; Δ*hfq*(*hfq*-pBBR), the *hfq* mutant containing *hfq*-pBBR complemented vector; Δ*hfq*(*chiA*-flag), the *hfq* mutant containing flag-tagged *chiA*; OH11(*chiA*-flag), the wild-type containing flag-tagged *chiA*.

**Table S1.** Mutant confirmation by PCR in this study.

**Table S2.** Primers used for in-frame deletion and complementation in this study.

**Table S3.** Three chitinase encoding genes in *Lysobacter enzymogenes*.

**Table S4.** Primers used for qRT-PCR or RT-PCR in this study.
