# Supplementary material for: Hfq regulates antibacterial antibiotic biosynthesis and extracellular lytic-enzyme production in Lysobacter enzymogenes OH11
Source: Microb Biotechnol. 2015 Feb 13;8(3):499–509. doi: 10.1111/1751-7915.12246 (PMC4408182; doi:10.1111/1751-7915.12246)
Supplement: Supplementary file 1 [file mbt20008-0499-sd1.doc]

**Hfq regulates antibacterial antibiotic biosynthesis and extracellular lytic-enzyme production in *Lysobacter enzymogenes* OH11**

Gaoge Xu^a^, Yuxin Zhao^a^, Liangcheng Du^c^, Guoliang Qian^a^*, Fengquan Liu^a, b^*

^a^ College of Plant Protection, Nanjing Agricultural University, Nanjing 210095, China/Key Laboratory of Integrated Management of Crop Diseases and Pests (Nanjing Agricultural University), Ministry of Education, China

^b^ Institute of Plant Protection, Jiangsu Academy of Agricultural Science, Nanjing 210014, P.R. China

^c^ Department of Chemistry, University of Nebraska-Lincoln, Lincoln, Nebraska 68588, United States

**Corresponding Author***: Fengquan Liu, fqliu20011@sina.com, +86-25-84396726

Guoliang Qian, glqian@njau.edu.cn, +86-25-84396109

**Running Title**: Hfq in *Lysobacter enzymogenes*

**Supporting Results**


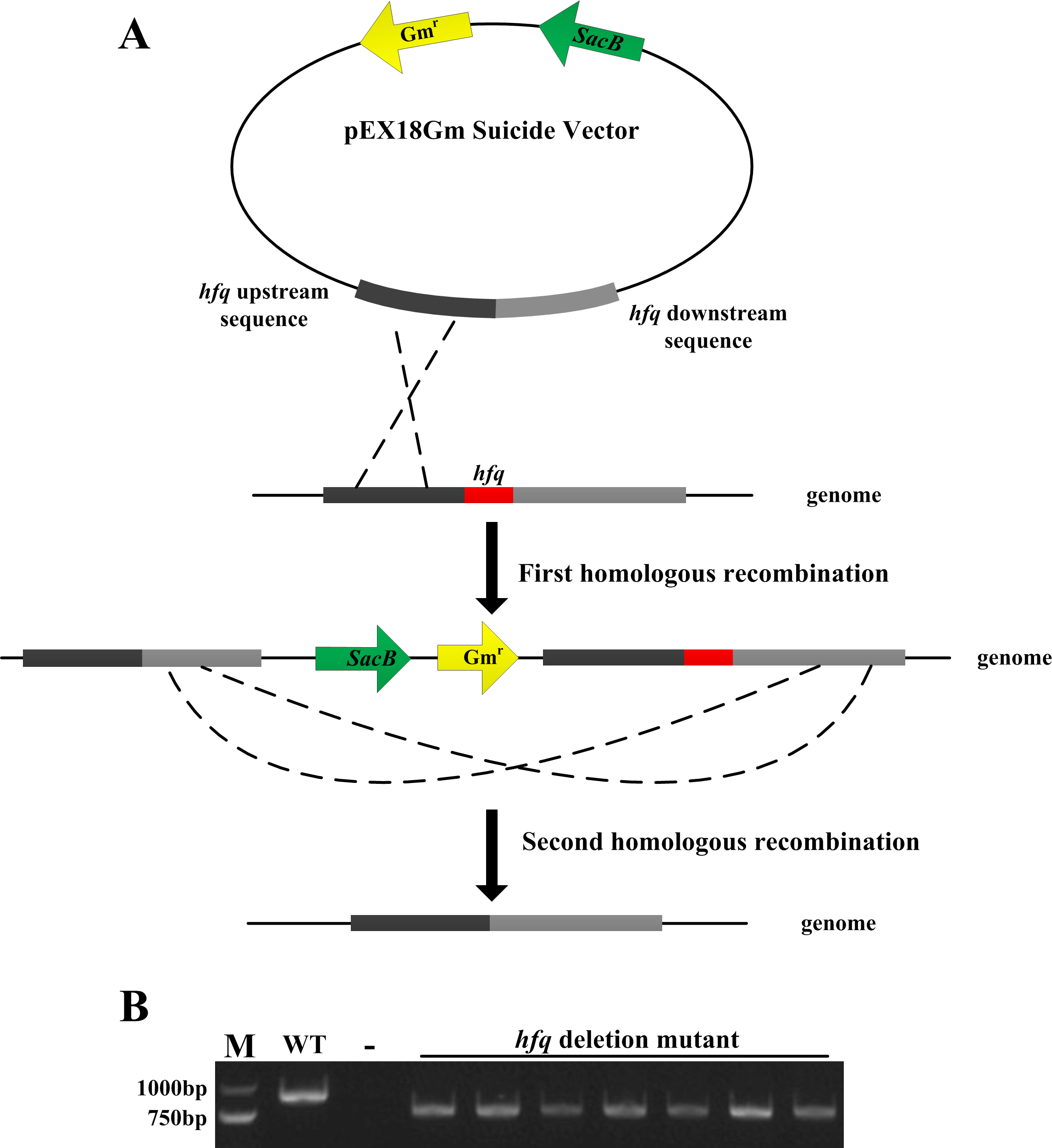


**Fig. S1** The scheme of the *hfq* mutant construction and molecular confirmation in *Lysobacter enzymogenes*. (A) Physical map of deletion mutant construction used in this study. The 364-bp (amplified by *hfq*-F1/R1) (Table S2) and 368-bp (amplified by *hfq*-F2*/*R2) (Table S2) DNA fragment of *hfq* was used as 5' and 3' fragment for homologue recombination, respectively. The internal 155-bp DNA fragment would be deleted in the *hfq* mutant. The primers *hfq*-F1/R2 (Table S2) was used for molecular confirmation of *hfq* mutant. (B) PCR verification of the *hfq* mutant. An 887-bp DNA fragment was amplified from the wild-type OH11 with the primers *hfq*-F1/R2, while only a 732-bp fragment was obtained from the *hfq* mutant with the same primers due to the deletion of internal 155-bp DNA fragment. ‘-’ in part B represent the blank control. This strategy was applied into the construction of other mutants of target genes in the present study (Table S1 and Table S2).


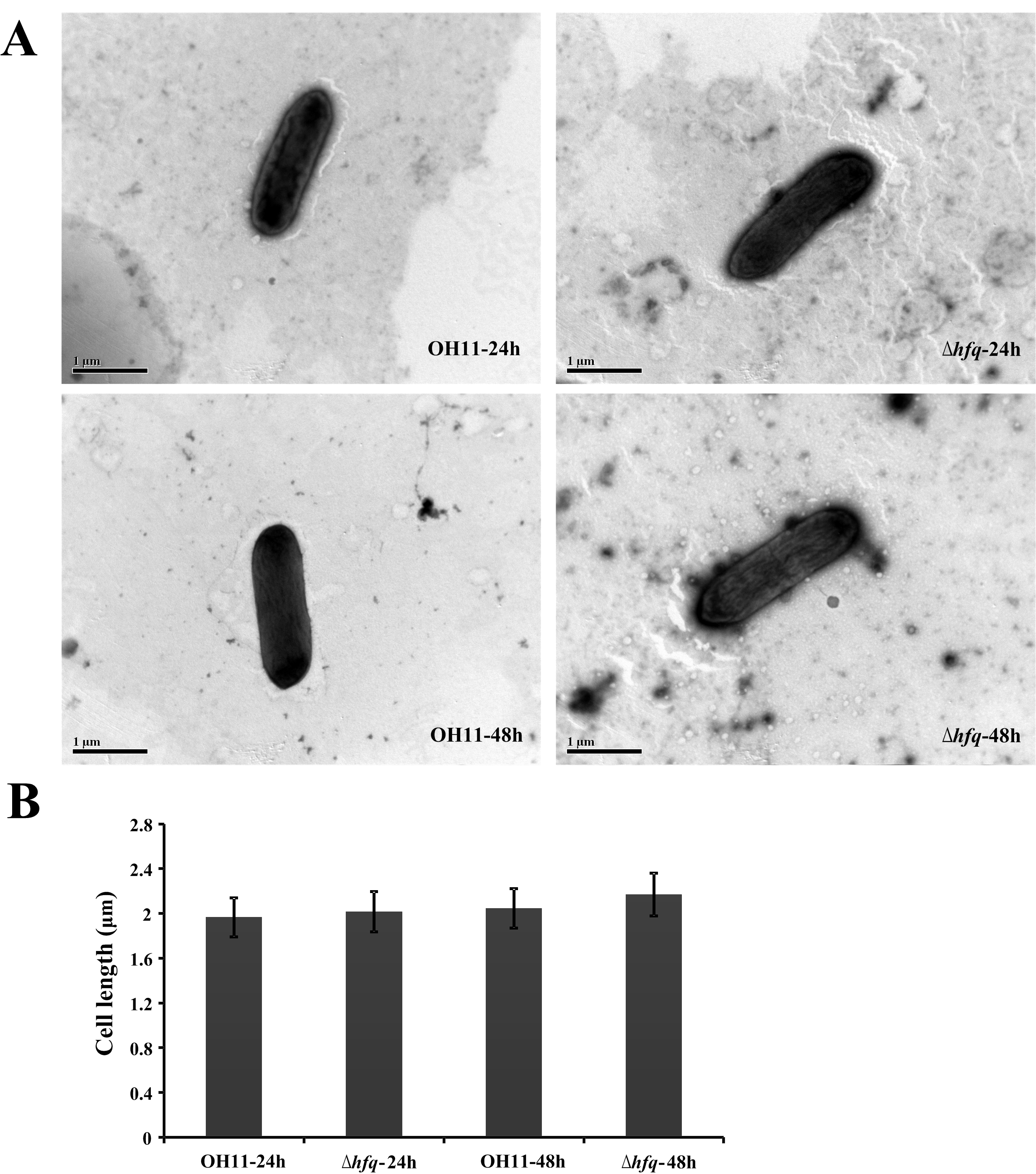


**Fig. S2** Comparison of the cell size between the wild-type strain and the *hfq* mutant of *Lysobacter enzymogenes*. (A) The representative result of cell size from three independent experiments between the wild-type strain (OH11) and the *hfq* mutant (Δ*hfq*) under electronic microscope at two selected time points (24 and 48 h after growth on solid 20% TSA medium). The scale bars represent 1 μm. (B) Statistic analysis of the cell size of the wild-type strain and the *hfq* mutant. In each technological repeat, at least 15 cells of each strain were selected for analysis. The experiment was performed three times. Each column indicates the mean of three biologically independent experiments. Vertical bars represent standard errors. No significant difference (P < 0.05; t-test) in cell size between the wild-type strain and the *hfq* mutant was found.


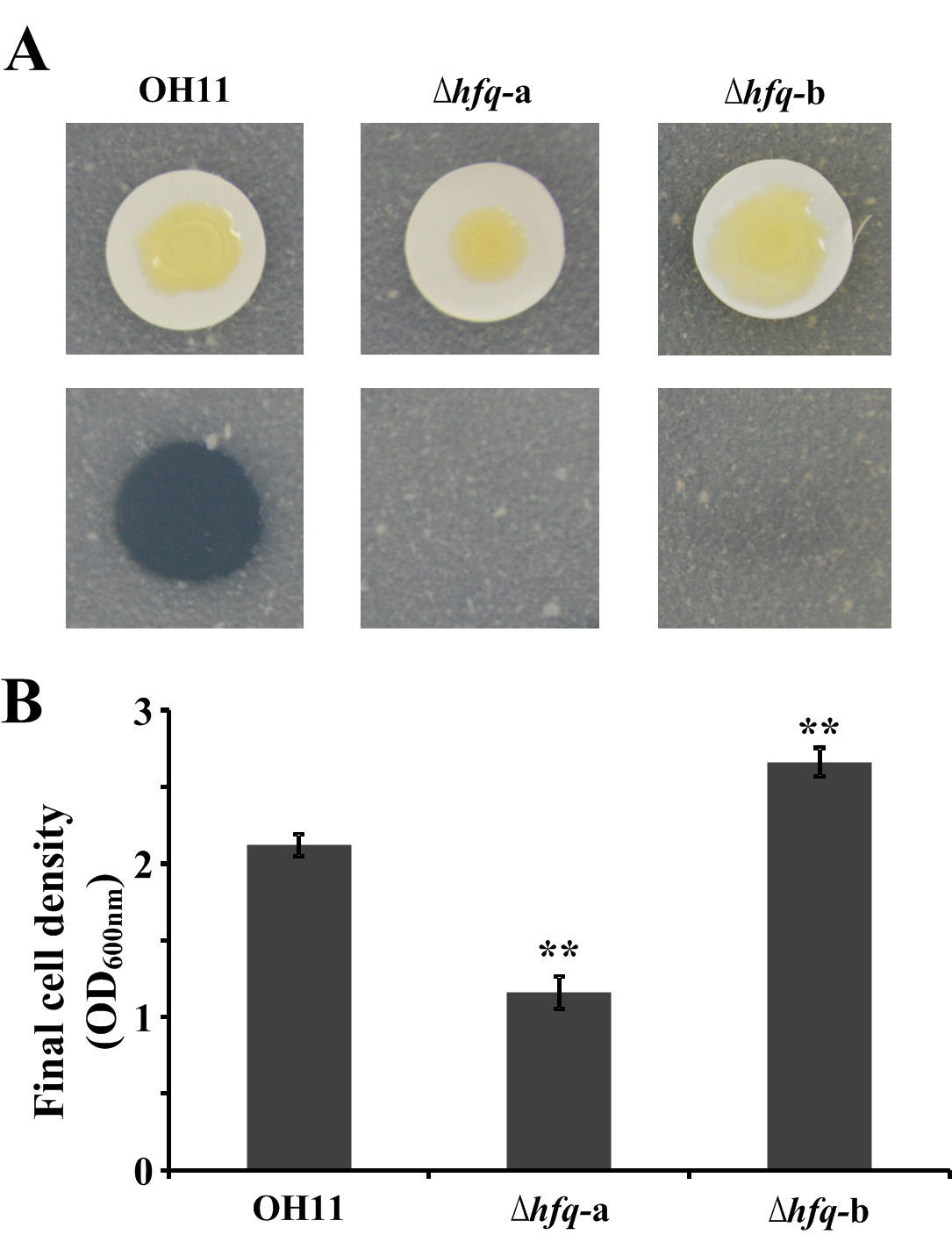


**Fig. S3** Further determination of extracellular chitinase activity between the wild-type strain and the *hfq* mutant of *Lysobacter enzymogenes* on solid medium. (A) The representative phenotype of extracellular chitinase activity between the wild-type OH11 and the *hfq* mutant from three independent experiments. (B) Quantitative analysis of cell density (OD_600nm_) of each strain on the surface of the filter membrane. Each column indicates the mean of three biologically independent experiments. Vertical bars represent standard errors. ‘**’ (p<0.01; t-test) above the bars indicate a significant difference between the wild-type strain and the *hfq* mutant. The initial inoculated cell concentration expressed by OD_600nm_ for the wild-type OH11, Δ*hfq*-a, Δ*hfq*-b was 2, 2, and 20, respectively.


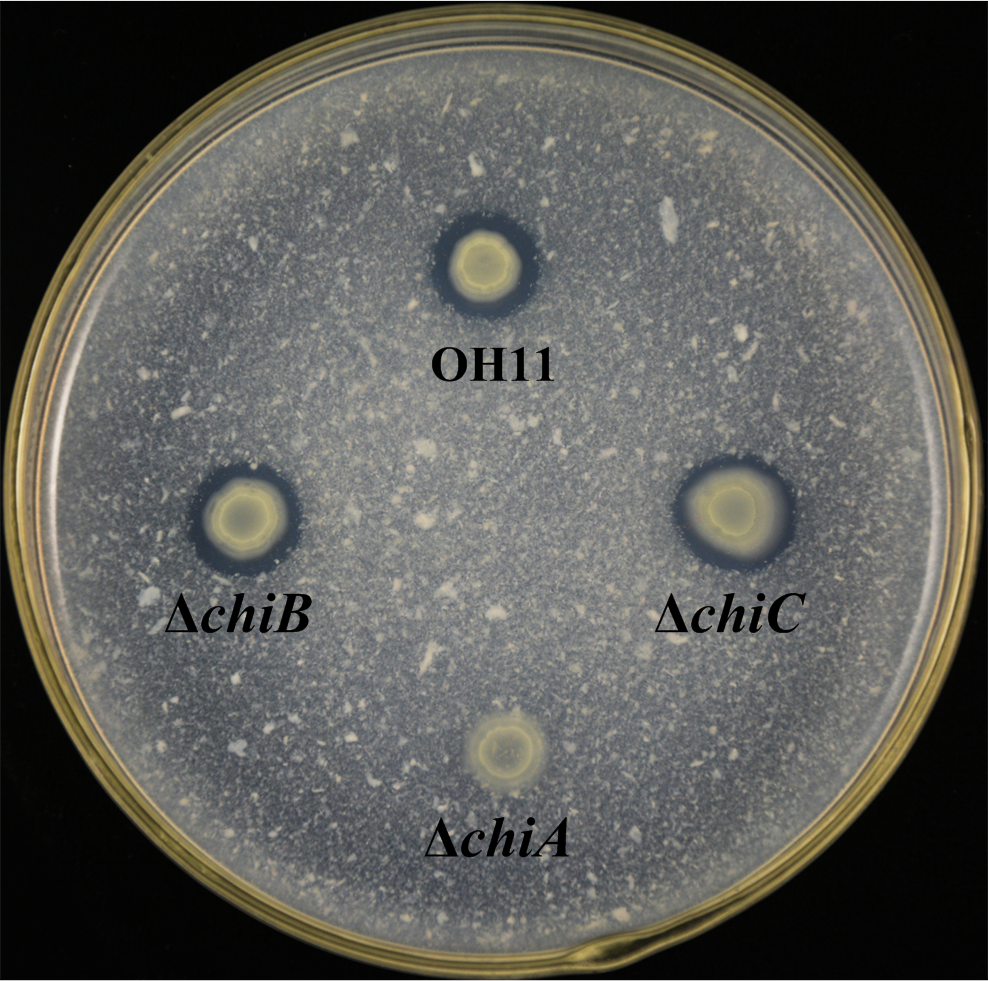


**Fig. S4.** Detection of the chitinase activity of three mutants of predicted chitinase synthesis genes (∆*chiA*, ∆*chiB*, ∆*chiC*) on chitin plate. Only the *chiA* mutant cannot hydrolyse chitin under the tested conditions. The gene information of *chiB* and *chiC* was provided in Table S3. The mutant construction and confirmation was provided in Table S1.


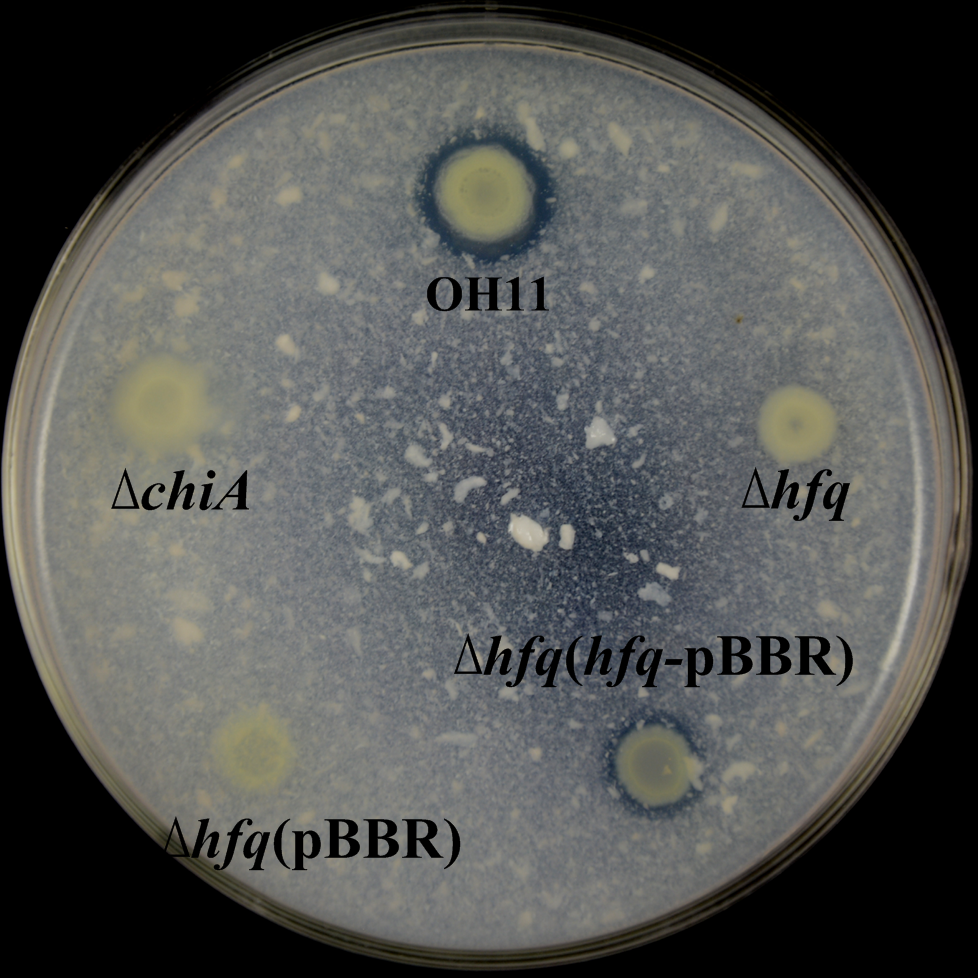


**Fig. S5.** Introduction of the broad-host-vector pBBR1-MCS5 containing *hfq* partially restored extracellular chitinase production of the *hfq* mutant in *Lysobacter enzymogenes*. OH11, the wild-type strain of *L. enzymogenes*; Δ*hfq*, the *hfq* deletion mutant; Δ*hfq*(pBBR), the *hfq* mutant containing the empty vector (pBBR1-MCS5); Δ*hfq*(*hfq-*pBBR), the pBBR1-MCS5 based *hfq* complemented strain. Fig. S5 is the representative result of three independent experiments.


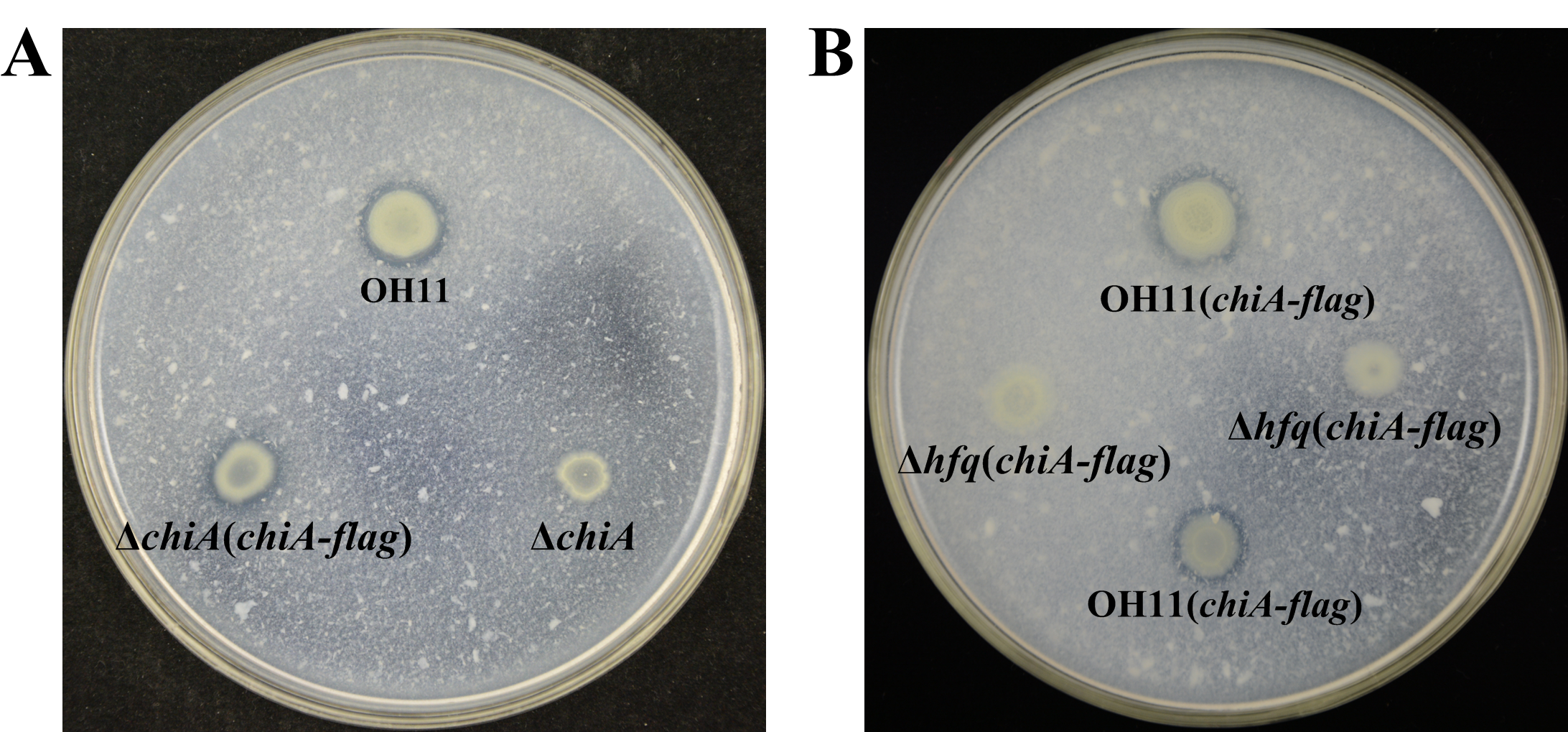


**Fig. S6.** Determination of extracellular chitinase activities of *Lysobacter* strains. (A) The construct of flag-tagged *chiA* restored the chitinase activity of the *chiA* mutant. (B) The *hfq* mutant containing the construct of flag-tagged *chiA* did not restore the chitinase activity. OH11, the wild-type strain of *L. enzymogenes*; ∆*chiA,* the *chiA* deletion mutant (Qian *et al*., 2012); ∆*chiB,* the *chiB* deletion mutant; ∆*chiC,* the *chiC* deletion mutant; ∆*chiA*(*chiA*-flag), the *chiA* mutant containing flag-tagged *chiA*; ∆*hfq*(pBBR), the *hfq* mutant containing an original pBBR1-MSC5 vector; ∆*hfq*(*hfq*-pBBR), the *hfq* mutant containing *hfq*-pBBR complemented vector; ∆*hfq*(*chiA*-flag), the *hfq* mutant containing flag-tagged *chiA*; OH11(*chiA*-flag), the wild-type containing flag-tagged *chiA*.

**Reference**

Qian, G. L., Wang, Y. S., Qian, D. Y., Fan, J. Q., Hu, B. S. and Liu, F. Q. (2012) Selection of available suicide vectors for gene mutagenesis using *chiA* (a chitinase encoding gene) as a new reporter and primary functional analysis of *chiA* in *Lysobacter enzymogenes* strain OH11. *World Journal Microbiology and Biotechnology* **28**(2): 549-557.
