# Supplementary material for: Hfq regulates antibacterial antibiotic biosynthesis and extracellular lytic-enzyme production in Lysobacter enzymogenes OH11
Source: Microb Biotechnol. 2015 Feb 13;8(3):499–509. doi: 10.1111/1751-7915.12246 (PMC4408182; doi:10.1111/1751-7915.12246)
Supplement: Supplementary file 2 [file mbt20008-0499-sd2.doc]

**Table S1 Mutant confirmation by PCR in this study**

| Mutant^a^ | Selected  Primers^b^ | Expected size from wild-type OH11 | Expected size from deletion mutant | PCR confirmation^c^ |
| --- | --- | --- | --- | --- |
| Part A  Δ*hfq*Δ*αlp* | *αlp*-F1/R | 1994 bp | 800 bp | 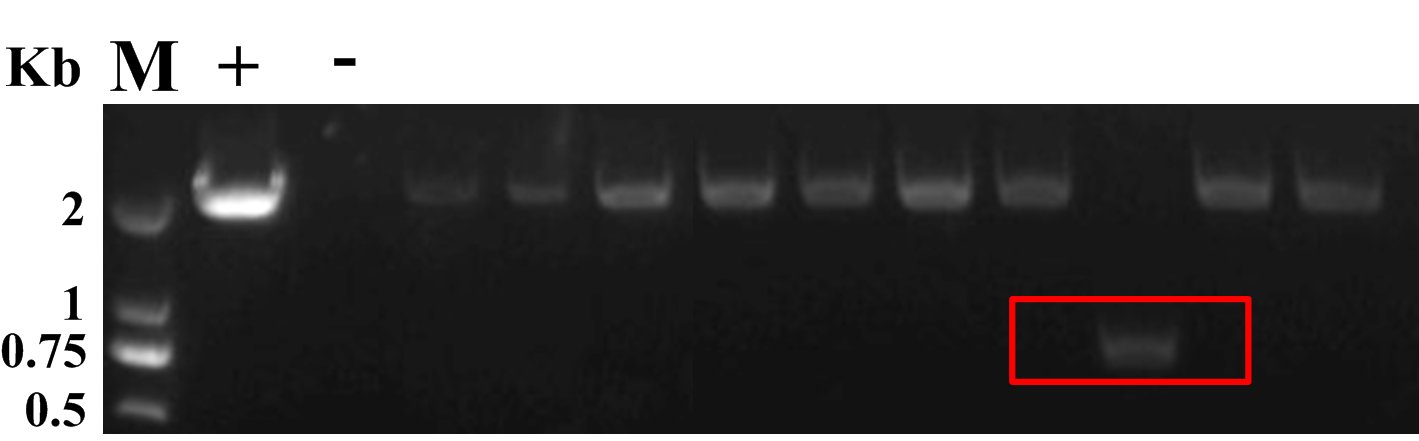 |
| Part B  Δ*chiB* | *chiB*-F1/R2 | 1873 bp | 993 bp | 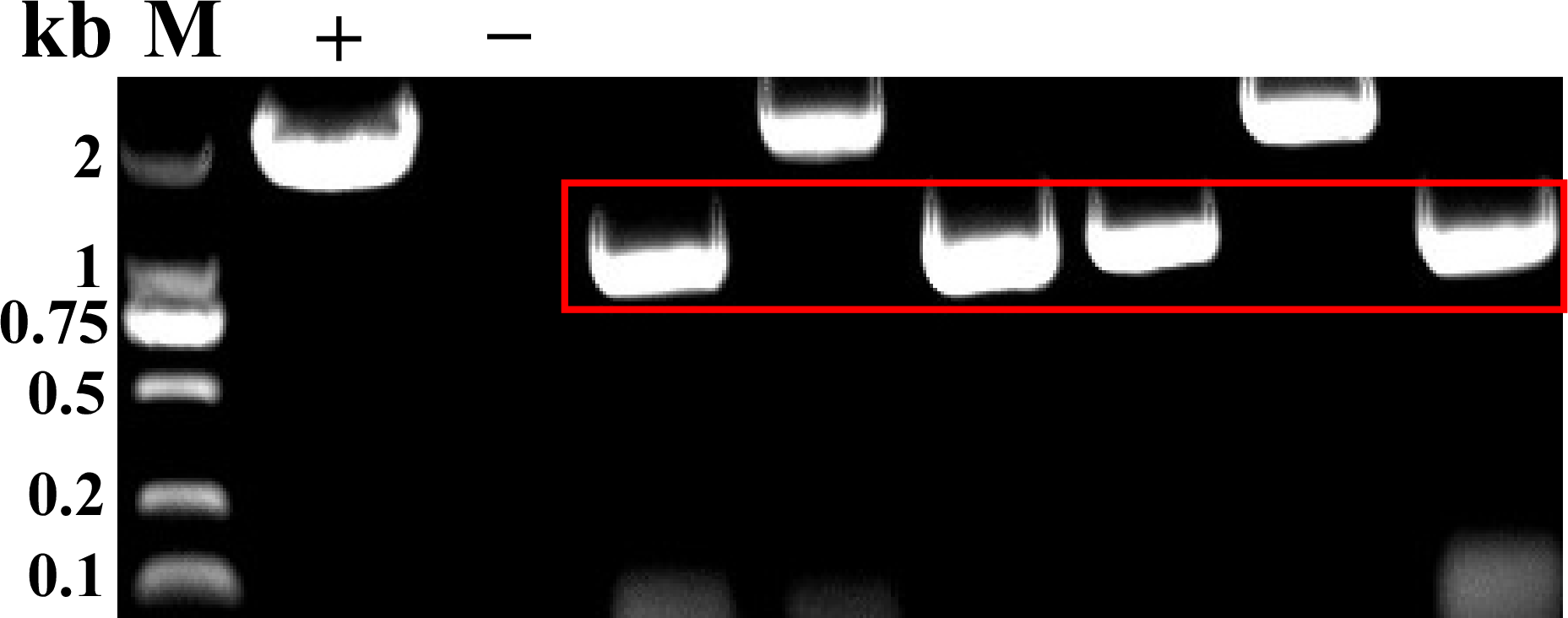 |
| Part C  Δ*chiC* | *chiC*- F1/R2 | 2281 bp | 1376 bp | 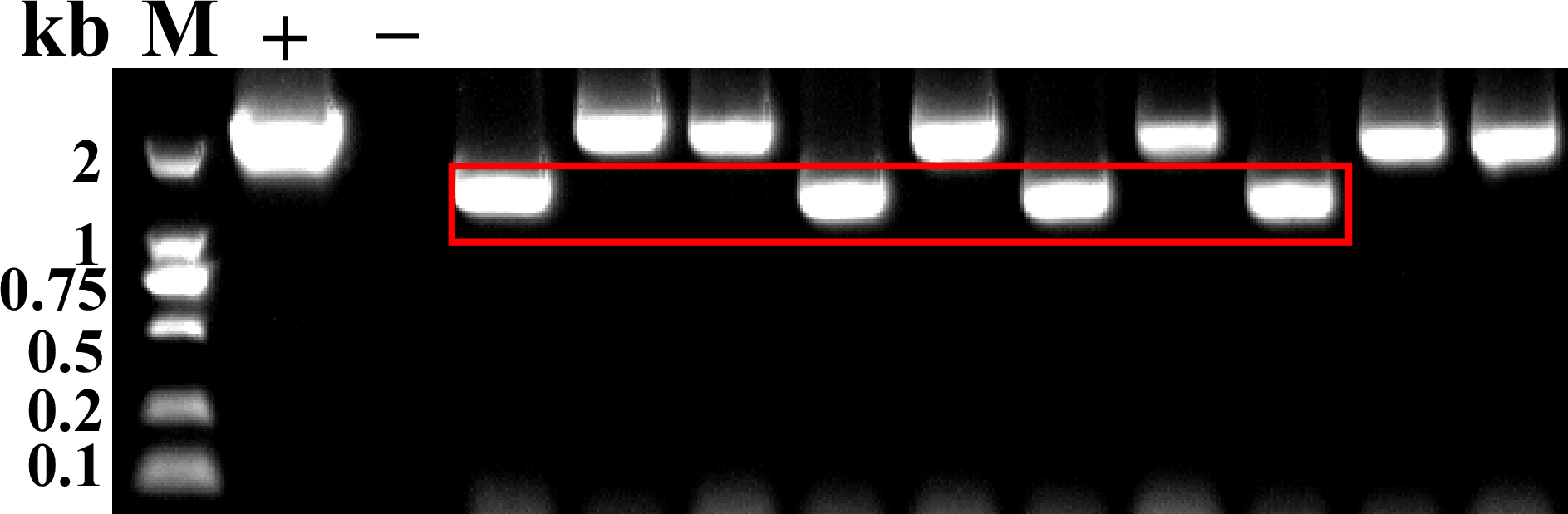 |

^a^ In part A, ‘+’ represents *hfq* mutant; in part B and C, ‘+’ represents the wild-type OH11. ‘-’ represents the blank control in all three parts.

^b^ Primers sequence used here were provided in Table S4.

^c^ Red box indicated the expected size from deletion mutant amplified by selected primers, respectively.
