# Supplementary material for: Hfq regulates antibacterial antibiotic biosynthesis and extracellular lytic-enzyme production in Lysobacter enzymogenes OH11
Source: Microb Biotechnol. 2015 Feb 13;8(3):499–509. doi: 10.1111/1751-7915.12246 (PMC4408182; doi:10.1111/1751-7915.12246)
Supplement: Supplementary file 3 [file mbt20008-0499-sd3.doc]

**Table S2 Primers used for in-frame deletion and complementation in this study**

| Primer | Sequence^a^ | Purpose |
| --- | --- | --- |
| *hfq*-F1 | CGGAATTCGAACTGGAGCACGCCCTGAG  (*Eco*R I) | To amplify a 364-bp upstream homologue arm of *hfq* |
| *hfq*-R1 | GCTCTAGAGCAGCTTGATGCCGTTGACC  (*Xba* I) |
| *hfq*-F2 | GCTCTAGAGGCAGCGACGAGAACGAATAAG  (*Xba* I) | To amplify a 368-bp downstream homologue arm of *hfq* |
| *hfq*-R2 | CCCAAGCTTAACGGGTGGTTGACCAGGATC  (*Hin*d III) |
| *hfq*-F | GAAGATCTTCCATCTTCGCCACCCGCCAGC  (*Bgl* II) | To amplify a 668-bp fragment containing intact *hfq* and its predicted promoter |
| *hfq*-R | GAAGATCTTCCCGGTTTCTCAATCGTCCGC  (*Bgl* II) |
| *chiB-*F1 | CCCAAGCTTCACAGCGGGGAGTTCGGGAT  (*Hin*d III) | To amplify a 468-bp upstream homologue arm of *chiB* |
| *chiB-*R1 | GGAATTCCATATGCTCAGGGTTCGGGTTCTCGG  (*Nde* I) |
| *chiB-*F2 | GGAATTCCATATGGGGCAACAACCTGGAGTGCT  (*Nde* I) | To amplify a 465-bp downstream homologue arm of *chiB* |
| *chiB-*R2 | GCTCTAGATCAAGCCGATCCGCCTGCAC  (*Xba* I) |
| *chiC-*F1 | CCCAAGCTTCAACGAGGAAGCAAGACATG  (*Hin*d III) | To amplify a 777-bp upstream homologue arm of *chiC* |
| *chiC-*R1 | GGAATTCCATATGGAGTGGATGGTCGGATGGGT  (*Nde* I) |
| *chiC-*F2 | GGAATTCCATATGCGCAGGAGAAACAGCAGACC  (*Nde* I) | To amplify a 599-bp downstream homologue arm of *chiC* |
| *chiC-*R2 | GCTCTAGAAGGTCGTGCTGGGGATGGCT  (*Xba* I) |
| *chiA-*F | CGGAATTCCGCCAGTTTCCCGATCCGAACG  (*Eco*RI) | To amplify a 2403-bp fragment containing intact *chiA and* its predicted promoter and the flag label |
| *chiA-*R | GCTCTAGAGCTCATTACTTATCGTCGTCATCCTTGTAATCCTTCAGCCCGTCGTCGAT  (*Xba* I) |
| *αlp-*F1 | CGGGATCCCGCGCGCATCGGCTCGATCGCT  (*Bam* HI) | To verify the *hfq* complemented strain |
| *αlp-*R2 | CCCAAGCTTGGGGTCCTGTTCGGCCTGTTCTA  (*Hin*d III) |

^a^ Restricted digestion enzyme site was underlined.
