# Supplementary material for: Hfq regulates antibacterial antibiotic biosynthesis and extracellular lytic-enzyme production in Lysobacter enzymogenes OH11
Source: Microb Biotechnol. 2015 Feb 13;8(3):499–509. doi: 10.1111/1751-7915.12246 (PMC4408182; doi:10.1111/1751-7915.12246)
Supplement: Supplementary file 4 [file mbt20008-0499-sd4.doc]

**Table S3 Three chitinase encoding genes in *Lysobacter enzymogenes***

| Gene ID | Amino Acids (aa) | Identity (%) | e-value | Annotation and Accession |
| --- | --- | --- | --- | --- |
| *lysE3059*  (*chiA*) | 675 | 77 | 0 | Chitinase A (*Stenotrophomonas maltophilia*)  gb AAB70917.1 |
| *lysE4952*  (*chiB*) | 318 | 63 | 1e-83 | Chitinase (*Streptomyces niveus*)  WP_023536568.1 |
| *lysE4235*  (*chiC*) | 377 | 56 | 1e-73 | Chitinase (*Xanthomonas hortorum*)  WP_023902365.1 |
