# Supplementary material for: Hfq regulates antibacterial antibiotic biosynthesis and extracellular lytic-enzyme production in Lysobacter enzymogenes OH11
Source: Microb Biotechnol. 2015 Feb 13;8(3):499–509. doi: 10.1111/1751-7915.12246 (PMC4408182; doi:10.1111/1751-7915.12246)
Supplement: Supplementary file 5 [file mbt20008-0499-sd5.doc]

**Table S4 Primers used for qRT-PCR or RT-PCR in this study**

| Gene | Primer sequence for 5' end | Primer sequence for 3' end | Primer source |
| --- | --- | --- | --- |
| 16s rRNA | 5'-ACGGTCGCAAGACTGAAACT-3' | 5'-AAGGCACCAATCCATCTCTG-3' | Qian *et al*., 2013 |
| *αlp* | 5'-CACCATCACCGCCAAGAAC-3' | 5'-CAGTTGTTGCCGTTGGACTG -3' | This study |
| *hfq* | 5'-ATCCTTTCCTGAATGCGCTG-3' | 5'-GGTGGAAATGGCGTGCTTAT-3' | This study |
| *waps1* | 5'-CGTATCGGTGTGGGAGTTCT-3' | 5'-TGATCACATCGGCGAGATAG-3' | Zhang *et al*., 2014 |
